# A biomechanics study on ligamentous injury in anterior-posterior compression type II pelvic injury

**DOI:** 10.1186/s13018-020-02156-w

**Published:** 2021-01-11

**Authors:** Jianzhong Kong, Yupeng Chu, Chengwei Zhou, Shuaibo Sun, Guodong Bao, Yu Xu, Xiaoshan Guo, Xiaolong Shui

**Affiliations:** 1 Department of Orthopedics Surgery, The Second Affiliated Hospital and Yuying Children’s Hospital of Wenzhou Medical University, NO. 109, Xue Yuan West Road, Wenzhou, 325027 Zhejiang Province China; 2 Department of Orthopaedics, The Central Hospital of Wenzhou, NO. 252, Baili Road, Lucheng District, Wenzhou, 325000 Zhejiang China

**Keywords:** APC fracture, Restricted group, Ligament injury, Separation distance

## Abstract

**Background:**

Anterior-posterior compression (APC) type II pelvis fracture is caused by the destruction of pelvic ligaments. This study aims to explore ligaments injury in APC type II pelvic injury.

**Method:**

Fourteen human cadaveric pelvis samples with sacrospinous ligament (SPL), sacrotuberous ligament (SBL), anterior sacroiliac ligament (ASL), and partial bone retaining unilaterally were acquired for this study. They were randomly divided into hemipelvis restricted and unrestricted groups. We recorded the separation distance of the pubic symphysis and anterior sacroiliac joint, external rotation angle, and force when ASL ruptured. We observed the external rotation damage to the pelvic bone and ligaments.

**Result:**

When ASL failed, there was no significant difference in pubic symphysis separation (28.6 ± 8.4 mm to 23.6 ± 8.2 mm, *P* = 0.11) and anterior sacroiliac joint separation (11.4 ± 3.8 mm to 9.7 ± 3.9 mm, *P* = 0.30) between restricted and unrestricted groups. The external rotation angle (33.9 ± 5.5° to 48.9 ± 5.2°, *P* < 0.01) and force (553.9 ± 82.6 N to 756.6 ± 41.4 N, *P* < 0.01) were significantly different. Pubic symphysis separation between two groups ranged from 14 to 40 mm. In the restricted group, both SBL and SPL were injured. SPL ruptured first, and then SBL and the interosseous sacroiliac ligament were damaged while the posterior ligament remained unharmed. In the unrestricted group, interosseous sacroiliac ligament and posterior sacroiliac ligaments were damaged, while SBL and SPL were not. When the ASL, SBL, and SPL all failed, pubic symphysis and anterior sacroiliac joint separation between two groups increased significantly (from 28.6 ± 8.4 to 42.0 ± 7.6 mm, 11.4 ± 3.8 to 16.7 ± 4.2 mm respectively, all *P* < 0.05).

**Conclusion:**

Pelvic external rotation injury is either hemipelvic restricted or unrestricted, which can result in different outcomes. When the ASL ruptures, the unrestricted group needs greater external rotation angle and force, without SBL or SPL injury, while both SBL and SPL were injured in another group. When ASL fails in two groups, pubic symphysis separation fluctuates considerably. Finally, when the ASL ruptures, SBL and SPL may be undamaged.

## Introduction

Pelvic fractures occur frequently and can be complicated by multiple traumas [1–3]. The anterior and posterior compression (APC) pelvic injury, a classic type also known as external rotation injury, is considered to be caused by the destruction of pelvic ligaments. On the basis of the anatomical and biomechanical research, the anterior structures, including the pubic symphysis and the pubic rami, contribute approximately 40% to the stability of the pelvis. The remaining 60% of pelvic stability is derived from the posterior structures, including the sacroiliac joint [4]. APC type II fracture is the intermediate type between stable and unstable external rotation pelvic fracture, the mechanism of which is still controversial [5, 6].

Thus, the injury mechanism and classification of APC type II need further refinement. There have been some biomechanical articles about APC pelvic fracture treatment [7, 8]; however, there are few about ligamentous injury in APC pelvic fracture. Toward this end, we biomechanically modeled the pelvic external rotation injury (APC type II pelvis fracture) to explore pelvic ligamentous injury and the separation distance of the pubic symphysis and sacroiliac joint when anterior sacroiliac ligament (ASL) ruptured. In addition, when the ASL, sacrotuberous ligament (SBL), and sacrospinous ligaments (SPL) all failed, we modeled bone and ligament injury of the anterior and posterior pelvic rings.

## Materials and methods

### Experimental materials

#### Main experimental instruments

ElectroForCeR 3510 biomechanical testing machine (Bose, MA, USA), WinTest control software, which is used for controlling testing machine and collecting high-precision data; motion dynamic motion capture system, which can capture pelvis rotation accurately; real-time video system; portable C-arm fluoroscopy and X-ray machine (provided by the Biomechanics Laboratory, Anatomy Teaching and Research Department, Nanfang Medical University); Vernier caliper; pelvic-embedded fixator; electric drill and Kirschner wires, hacksaw, scalpel, bone rongeur, periosteal stripper, vise, and protractor.

#### Main experimental reagents

This includes embedded specimens of denture powder (PMMA, type II) and denture water (Shanghai coral chemical plant). The volume ratio is 2:1 powder and water for 30 min at room temperature.

#### Experiment specimens

**Fig. 1:**
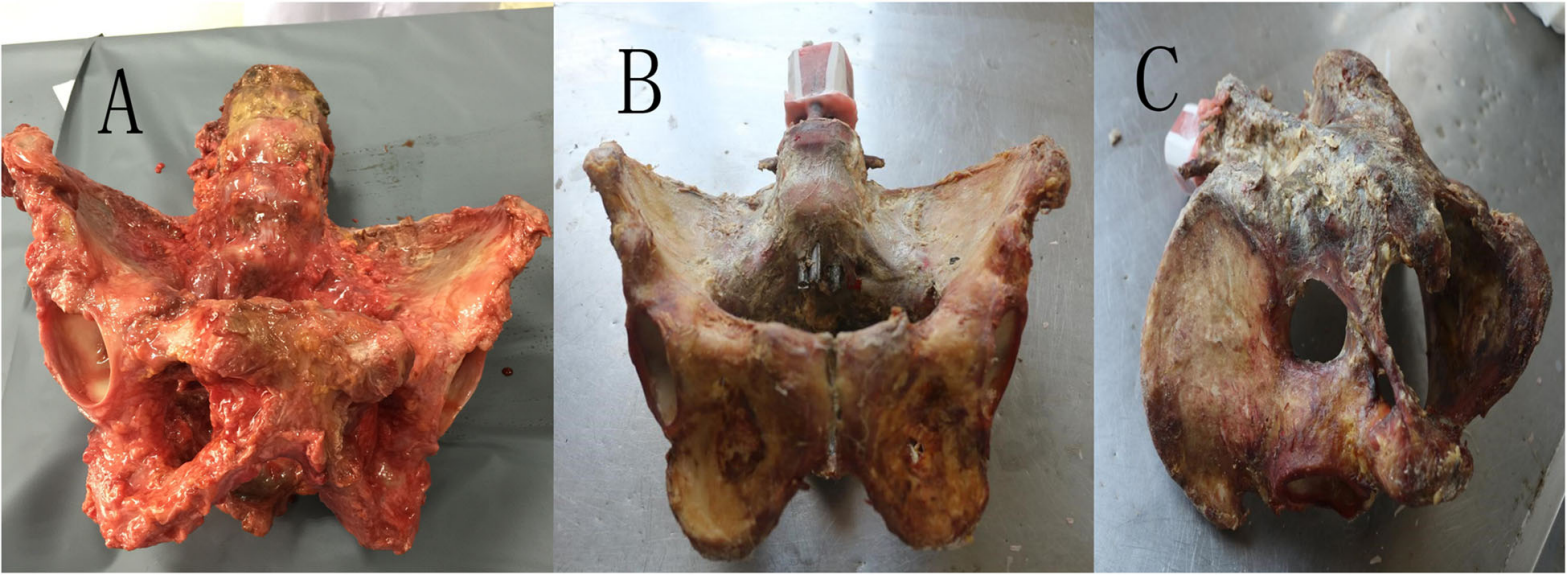
**a** Pelvic specimens from fresh cadavers. **b**, **c** Anterior and posterior view of the immobilized pelvic specimen

Inclusion criteria: (1) specimens were 18 years of age or older; (2) not associated with pelvic tumor, previous fracture, or sacroiliac joint fusion; and (3) by naked eye and X-ray plain film observation, specimens were of bilateral symmetry. Consequently, 14 fresh adult cadaver specimens without anti-corrosive treatment were collected for this study (5 females and 9 males, all of the specimens are from the Department of Human Anatomy, Nanfang Medical University). We intercept the cadaver specimen and preserved waist 5 and symphysis pubis, leaving the complete pelvic part retaining bone and ligaments (pubic symphysis ligament, SPL, SBL, anterior and posterior sacroiliac ligaments, and sacroiliac interosseous ligament) were numbered 1-14. The specimens were soaked in normal saline and wrapped in double-layer plastic bags to prevent drying and dehydration when not used. They were cryopreserved at −20 °C and equilibrated at room temperature 12 h before the experiment (Table 1, Fig. 1).

**Table 1:** Characteristics of the specimens and grouping result

Number	Female	Age	Separation distance of symphysis pubis (mm)	Separation distance of the sacroiliac joint (mm)	Time from anatomy to experiment (day)	Group	Rotation side
1	F	29	3.7	0.5	5	R	L
2	M	36	5.9	0.6	5	UR	R
3	M	48	4.7	0.5	4	R	L
4	F	52	3.0	0.4	4	UR	R
5	F	53	4.5	0.3	3	R	L
6	M	56	4.8	0.7	6	UR	R
7	M	37	4.9	0.5	4	R	L
8	M	45	6.0	0.6	3	UR	R
9	M	40	4.7	0.4	5	R	L
10	M	55	5.6	0.3	5	UR	R
11	F	60	3.5	0.8	4	R	L
12	M	39	4.2	0.5	6	UR	R
13	F	37	4.8	1.0	5	R	L
14	M	58	3.8	0.8	6	UR	R

*F* female, *M* male, *R* restricted group, *UR* unrestricted group, *L* left, *R* right

### Experiment method

**Fig. 2:**
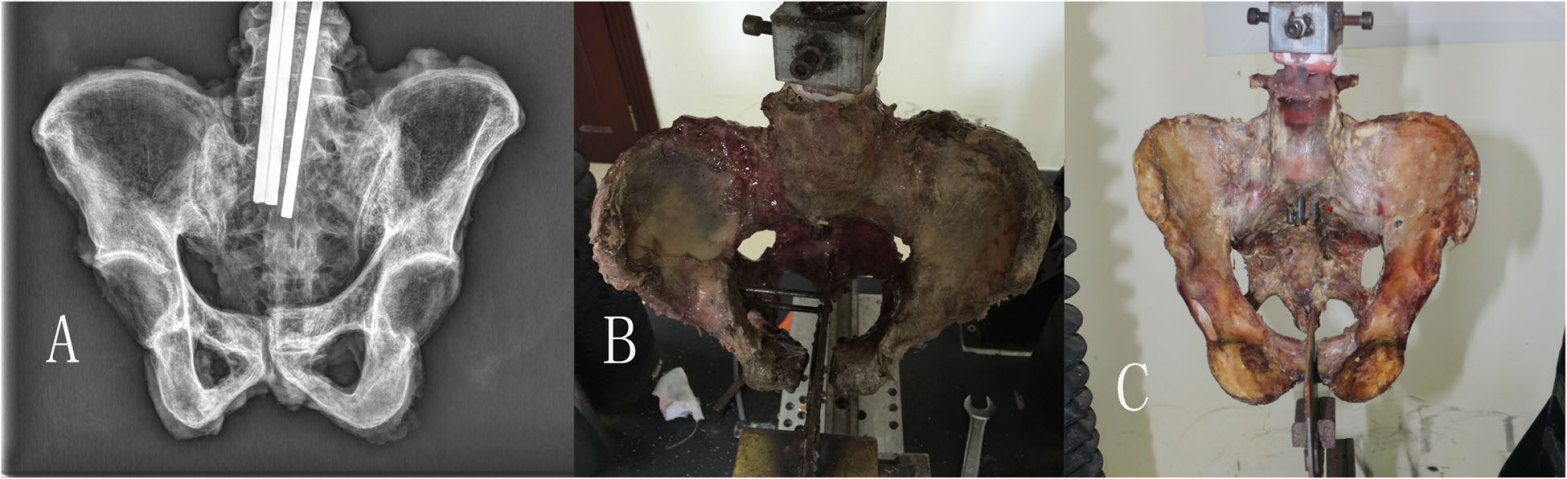
**a** X-ray image of the pelvic specimen before the experiment. **b**, **c** Restricted hemipelvis model and unrestricted hemipelvis model, respectively

The exact mechanism of pelvic external rotation injury in vivo is not clear, and the specific steps of the original Tile test were also not clear. Therefore, we stimulated and modified two different APC injury models based on the articles reported in recent years [7], and randomly divided the specimens into two groups, and made two kinds of pelvic external rotation injury test models. One group was the hemipelvic restricted group (1, 3, 5, 7, 9, 11, 13); the one was the unrestricted group (2, 4, 6, 8, 10, 12, 14). In both the groups, 3-4 steel K-wires with a diameter of 1 cm were inserted into the pelvis from the middle of the fifth lumbar vertebrae to the center of the sacrum, and then the pelvis was embedded with a fixator to provide the axial load. To measure the separation distance of pubic symphysis when ASL ruptured, a 0.3-cm steel plate was inserted into pubic symphysis to maintain a 0.3-cm reference point to calculate separation distance. After the plate was fixed onto the test machine base, we used two screws to fix the pelvic acetabulum test side of the restricted group to restrict other directional movement with the exception of internal and external rotation. However, in the unrestricted group, we did not fix the pelvis with screws in order to allow vertical and sagittal movements (Fig. 2). The restricted group provided data on pure external rotation mode, while the unrestricted group provided a composite model—external rotation with flexion and extension displacement. We tested both models as it is not clear, which is closer to the typical APC injury. To exclude differences between the left and right pelvic sides, we also randomly selected half of the specimens to externally rotate on the right side and the other half on the left side. Finally, each pelvic specimen was designed with one side of the sacroiliac joint restricted and the other side unrestricted.

Next, we cut off the pubic symphysis from all fresh cadavers and fixed on a biomechanical machine. The pelvic specimen is fixed on a fixture that allows one side of the sacroiliac joint to be open when the hemipelvis is under external rotation, but the other side is in control. We simulated APC injury by setting the sacrum at the center and 250 nm as the maximum possible torque, and performed a pure external rotation of 2°/s. The rotational shift was measured with a rotary variable difference converter. The biomechanical machine recorded angular displacement at each torque and formed a torque curve. When the torque curve slipped sharply and suddenly, this indicated that the ASL ruptured or was seriously deformed. At this point, we observed whether the SPL and SBL were damaged by both visual inspection and video, and measured the separation distances of the pubic symphysis and anterior sacroiliac joint. Then, we continued to externally rotate the hemipelvis until both the SPL and SBL failed. At this point, we observed a separation between the pubic symphysis and anterior sacroiliac joint, and the posterior ligaments including the interosseous sacroiliac ligament and posterior sacroiliac ligament.

### Observation index

1. When the ASL ruptured, we recorded the separation distance of pubic symphysis and anterior sacroiliac joint, the external rotation angle and force, and observed whether the SPL and SBL were damaged.
2. When the ASL, SPL, and SBL all ruptured, we observed the bone and posterior ligaments change and recorded the external rotation angle, force, and relevant separation distances.

Statistical analysis was performed using SPSS22.0. We analyzed the data using paired sample *t* tests. Differences were considered to be statistically significant if *P* < 0.05.

## Results

**Fig. 3:**
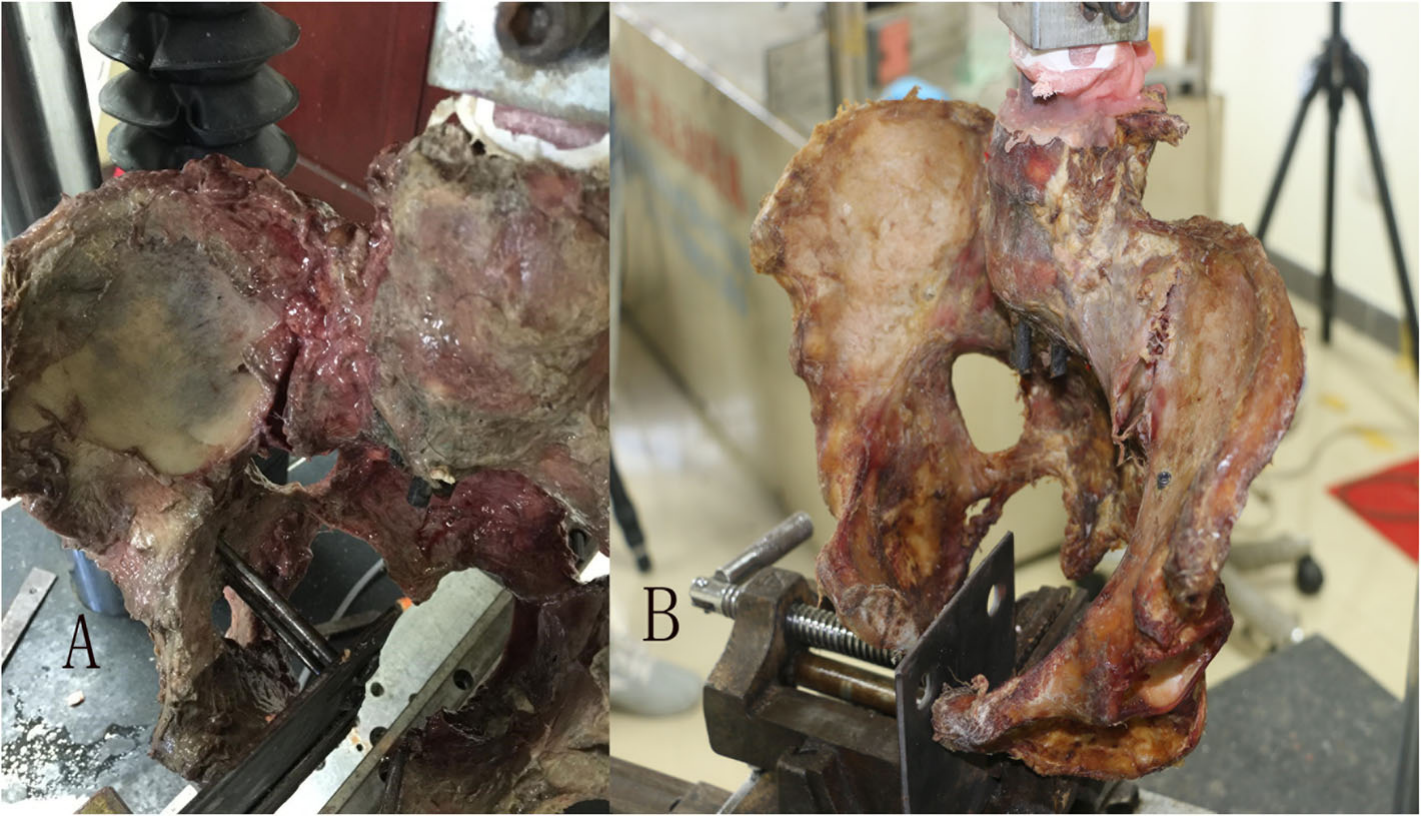
**a**, **b** Samples in restricted group and unrestricted group have anterior sacroiliac ligament failed

**Fig. 4:**
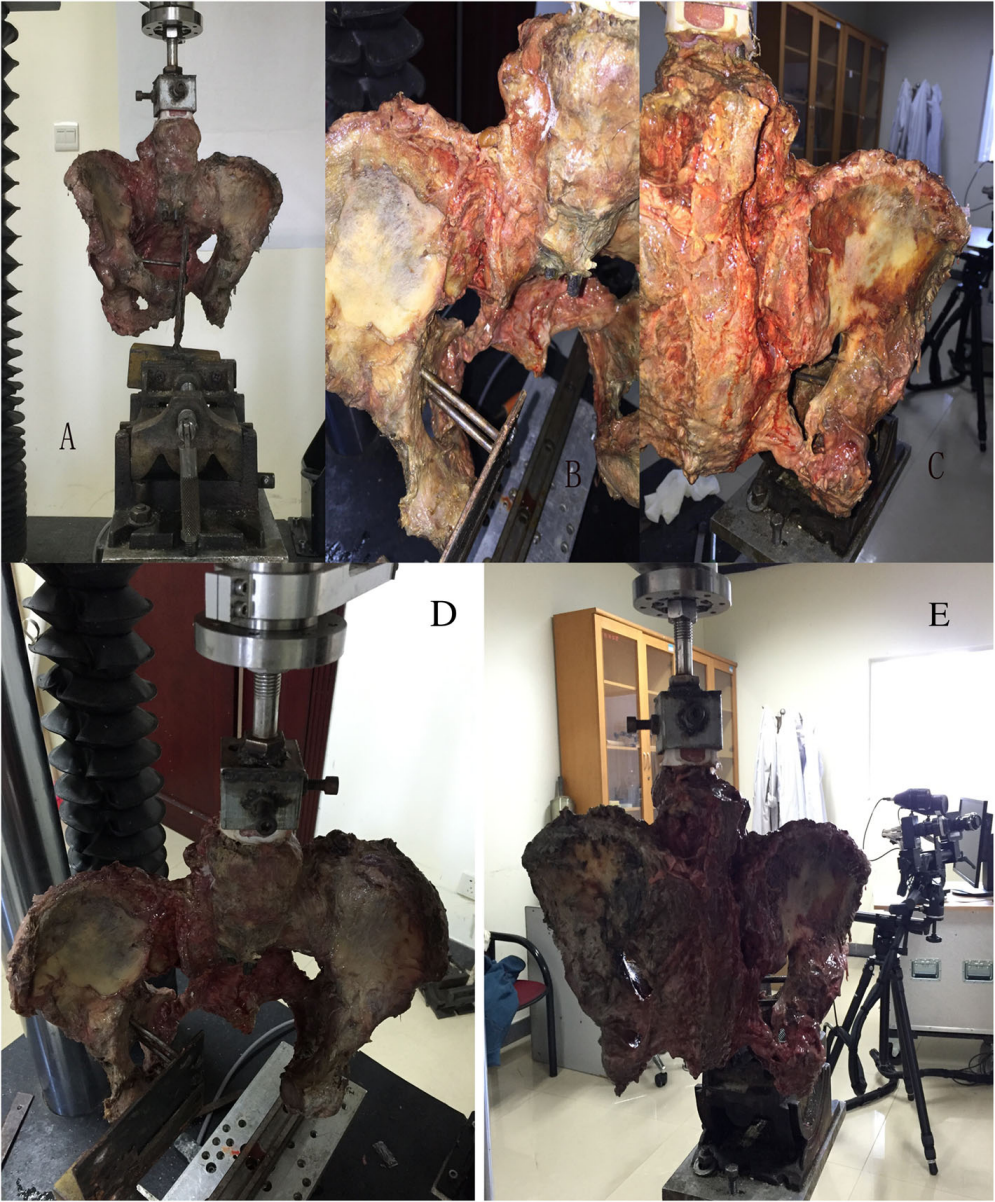
**a**-**c** In the restricted group, the anterior sacroiliac ligament fails and sacrospinous ligament rupture. **d**, **e** In the restricted group, the anterior sacroiliac ligament fails, both of sacrospinous ligament and sacrotuberous ligament rupture

When the ASL ruptured (Fig. 3), the mean separation distance of pubic symphysis and anterior sacroiliac joint between two groups was 28.6 ± 8.4 mm to 23.6 ± 8.2 mm and 11.4 ± 3.8 mm to 9.7 ± 3.9 mm, respectively (*P* > 0.05 for all). Mean external rotation angle and force between the two groups were 33.9 ± 5.5° to 48.9 ± 5.2° and 553.9 ± 82.6 N to 756.6 ± 41.4, respectively (*P* < 0.01 for all). The unrestricted group showed greater external rotation angle and force compared with the restricted group (Table 2). In addition, the restricted group was associated with SPL and SBL injury in all samples. In the restricted group, two samples had SBL ruptured and three specimens had complete SPL ruptured (Fig. 4). In addition, two specimens suffered complete SPL and SBL rupture simultaneously. In contrast, no distinct SBL or SPL injury was observed in the unrestricted group (Table 3).

**Table 2:** When anterior sacroiliac ligaments rupture, comparison of relevant variables between two groups

Variable	Restricted group	Unrestricted group	*T*	*P* value
Separation distance of symphysis pubis (mm)	28.6 ± 8.4	23.6 ± 8.2	1.871	0.11
Separation distance of anterior sacroiliac joint (mm)	11.4 ± 3.8	9.7 ± 3.9	1.137	0.30
External rotation angle (°)	33.9 ± 5.5	48.9 ± 5.2	−5.79	< 0.01
External rotation force (N)	553.9 ± 82.6	756.6 ± 41.4	−6.125	< 0.01

**Table 3:** When anterior sacroiliac ligaments rupture, comparisons of sacrotuberous ligaments, and sacrospinous ligaments between two groups

	Restricted group							Unrestricted group						
	1	3	5	7	9	11	13	2	4	6	8	10	12	14
Sacrotuberous ligament	Y	Y	F	Y	Y	F	Y	N	N	N	N	N	N	N
Sacrospinous ligament	Y	F	F	Y	Y	F	Y	N	N	N	N	N	N	N

*N* normal; *Y* ligament is injured (including ligament is prolonged, torn, and the tension cannot be restored obviously); *F* ligament ruptures completely

**Fig. 5:**
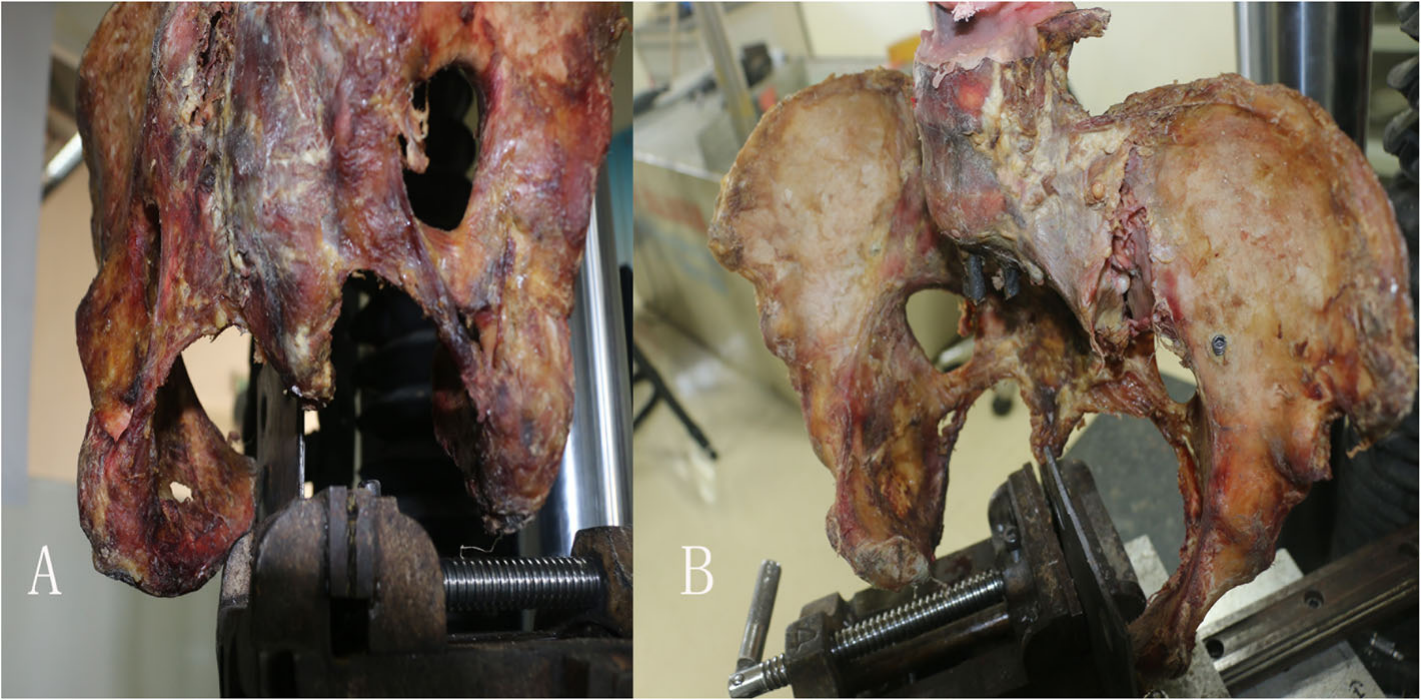
**a**, **b** In the unrestricted group, sample is under extreme external rotation force, no obvious sacrospinous ligament or the sacrotuberous ligament injury is seen, but the posterior sacroiliac ligament injury is seen

**Fig. 6:**
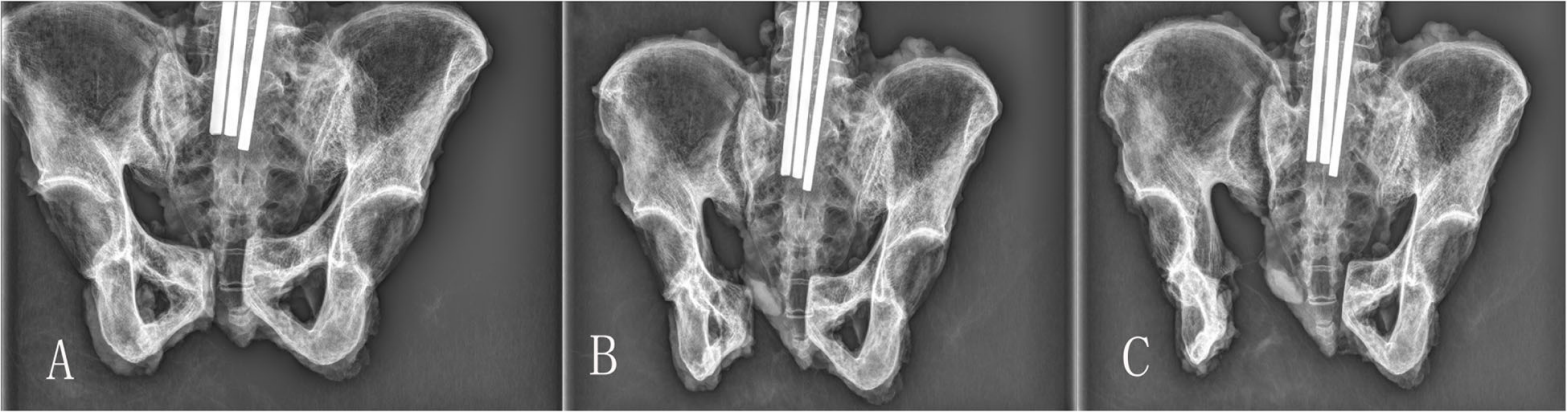
**a**-**c** In the restricted group, the X-ray images are taken when anterior sacroiliac ligament fails, sacrospinous ligament, and sacrotuberous ligament rupture in turn, which shows that the pubic symphysis separation increases distinctly

After the ASL ruptured, we continued the external rotation. In the unrestricted group when samples underwent extreme external rotational force, there was still no SBL or SPL injury. However, the interosseous sacroiliac ligament and posterior sacroiliac ligament injury with a slight sagittal rotation displacement of the sacroiliac joint occurred (Fig. 5). In the restricted group, with the external rotation force continuing, SPL and SBL ruptured in turn. When both of the two ligaments ruptured, the interosseous sacroiliac ligaments were injured as well, but the posterior sacroiliac ligaments were not. When three ligaments all ruptured, the separation distances of the pubic symphysis and sacroiliac joint were 42.0 ± 7.6 mm and 16.7 ± 4.2 mm, respectively, which increased evidently when compared with the distances when only ASL ruptured (28.6 ± 8.4 mm and 11.4 ± 3.8 mm, both *P* < 0.05). These data suggest that the pelvic ring was more unstable (Table 4; Fig. 6, sample from the restricted group).

**Table 4:** When the three ligaments all fail, compared symphysis pubis separation to the separation when anterior sacroiliac ligament barely fails

Variable	Restricted group^1^	Restricted group^2^	*T*	*P* value
Separation distance of symphysis pubis (mm)	28.6 ± 8.4	42.0 ± 7.6	2.836	0.030
Separation distance of anterior sacroiliac joint (mm)	11.4 ± 3.8	16.7 ± 4.2	2.880	0.028

^1^The separation distance when anterior sacroiliac ligament barely fails

^2^The separation distance when the anterior sacroiliac ligament, sacrotuberous ligament, and sacrospinous ligaments all fail

## Discussion

Much published research on pelvic anatomy, morphology, and biomechanics provides a good foundation for conducting a pelvic ligaments study. The ASL covers the front of the sacroiliac joint, which is a wide and thin fiber bundle. As the ligament structure is relatively weak, it has little impact on stabilizing the sacroiliac joint. The interosseous sacroiliac ligament is composed of many short and strong fiber bundles filling the irregular joint space at the upper back of the sacroiliac joint. The posterior sacroiliac ligament, also known as the dorsal sacroiliac ligament, divides into shallow and deep layers. The posterior sacroiliac ligament and the interosseous ligament constitute the sacroiliac ligament complex, which forms the main mechanical resistance at the back of the sacroiliac joint [6–16]. Tile et al. conducted mechanical experiments, which confirmed that if the posterior sacroiliac ligament complex remains intact, even if other pelvic ligaments ruptured, there would be no backward and up-down displacement of the hemipelvis [14]. However, the control of the control rotational force in the posterior sacroiliac ligament complex was poor. In our study, we design two different models with better control. Our pelvic samples are fixed on the biomechanical machine with K-wires or screws, and confirm the rotation of the sacroiliac joint on the testing side is the external rotation side.

In a 2002 study, Dujardin et al. discovered that the SPL and SBL played important roles in maintaining the stability of the sacroiliac joint. Cutting the two ligaments could significantly increase not only the angular displacement of the sacrum but also the vertical displacement [17]. SPL mainly controls the external rotation of the pelvic ring, while the SBL controls the vertical shear force acting on the semi-pelvic area. The two ligaments form a 90° angle to each other, similar to the cruciate ligament of the knee joint, and control pelvic vertical external force and external rotational force. These two ligaments can also strengthen the posterior sacroiliac ligament. Vukicevic et al. discovered that the two ligaments had no preventive effect on pelvic movement, and their removal also had no effect on sacroiliac joint movement [18].

APC pelvic injury is considered a typical pelvic ligament injury, which is classified into three types. In type I, one or both sides of pubic branches fracture or separation distance of pubic symphysis is less than 2.5 cm, and/or slight separation of anterior sacroiliac joint, but the anterior and posterior ligaments are intact; type II, the pubic symphysis separates more than 2.5 cm, the ASL, SPL, and SBL rupture, and the sacroiliac joint separates slightly; type III, the hemipelvis separates completely, but there is no longitudinal displacement. The anterior and posterior ligaments are injured at the same time, and the sacroiliac joint is separated [6, 18]. Among the three types, APC type II is the intermediate type, between stable and unstable, but there is controversy regarding the ligaments injured. Thus, it is necessary to further study the mechanism of APC pelvic ligament injury.

For APC type I fracture, ASL is considered to be intact and conservative treatment can be taken. While for APC type II fracture, ASL is injured and operative treatment is required [19]. It is accepted that when the ASL ruptures, the separation distance of pubic symphysis must be more than 2.5 cm. Of note, the 2.5 cm distance is used in the Tile classification system to distinguish type B1 pelvic fracture as well [18]. According to our experimental outcome, when APC type II fracture is associated with ASL failure, the mean separation distance is 2.38 cm, which is close to 2.5 cm. However, the distance of each specimen shows great differences, ranging from 1.4 to 4.0 cm. This result indicates that the ASL is likely to rupture when the displacement is more than 4.0 cm, but it may not happen between 1.4 and 4.0 cm.

In addition, we find that the SPL and SBL do not always rupture completely in APC type II fractures. In the unrestricted group, all of the pelvic floor ligaments are intact, while in the restricted group, only two samples rupture completely out of seven. Slocumn and Terry reported that the sacrum was easier to rotate and bend after removal of SPL and SBL [20]. Edeiken et al. reported that the sacrum was unstable when the two ligaments were torn [21]. In the unrestricted group, our results show that rotational force enables the pelvis to rotate around the sacroiliac joint axis without necessarily damaging the two ligaments. Thus, our research provides much supplementary content to APC pelvic injury. The two ligaments can make the hemipelvis open by external rotation force, guiding the hemipelvis to move downward. APC type II injury with intact SPL and SBL is a relatively stable pelvic injury, which can be treated without surgical intervention; however, we need more clinical outcome measures to confirm this finding.

In summary, we found (1) pelvic external injury can be divided into two situations—restricted and unrestricted. It is not certain which situation is closer to reality, but the results are distinctly different. When the ASL ruptures, the angle and force of external rotation between the restricted group and the unrestricted group are significantly different. In the unrestricted group, when the ASL ruptures, no SPL or SBL damage is observed, while the outcome in the restricted group is the opposite. According to the experimental results and references [16, 17], we consider that SPL and SBL are used to enhance the stability of the pelvic floor and can be destroyed by vertical and sagittal rotations. As we find in the restricted group, the deformation force requires a vertical component, which is related to the axis of the sacroiliac joint. We predict that in the unrestricted group, the pelvis can rotate around the axis of the sacroiliac joint without damaging the two ligaments. In the restricted group rotation and vertical displacement are eliminate, which results in the two ligaments being easily injured. (2) When ASL fails, the mean pubic symphysis distance is 2.38 cm, which is close to 2.5 cm, but the specific data of each sample is various, ranging from 1.4 to 4.0 cm. Doro CJ et.al also were skeptical about the assumption that anterior sacroiliac ligament injury based on symphyseal diastasis of 2.5 cm [22]. Two studies showed that 27 to 50% of APC I injuries exceeded 2.5 cm during physical examination performed with the patients under anesthesia [23, 24]. Thus, we postulate that 2.5 cm is not the critical standard to distinguish the APC type I from type II pelvic injury. We suggest that clinicians should be cautious in making clinical treatment decisions when the anterior sacroiliac and pelvic ring ligaments are damaged, and the pubic symphysis displacement is more than 2.5 cm. Although static and dynamic pelvic imaging can provide useful evidence, we should not make absolute clinical judgment on the ASL injury based on the separation distance of pubic symphysis, unless the distance is more than 4 cm; (3) when the ASL is destroyed, we do not observe the inevitable destruction of the pelvic floor ligaments (SPL and SBL). Among all the samples, only two cases show complete SPL and SBL rupture, and none of the samples in the unrestricted group show pelvic floor ligament rupture. However, we did confirm that when both ligaments fail, the pelvic injury is more serious, which has effect on the interosseous sacroiliac joint ligament and the stability of the posterior pelvis. Gary J L et al. also considered that rupture of the two ligaments, and/or pelvic floor musculature can imply a more severe and unstable injury pattern than that indicated by rupture of the ASL alone [10]. When extreme external rotation force is performed in the unrestricted group, the interosseous sacroiliac ligament and posterior sacroiliac ligament torsion damage appears first. This affects the stability of the whole hemipelvis, leading to vertical and sagittal rotational instability, followed by pelvic floor ligament rupture. According to this experiment, we consider that the pelvic external rotation injury is not simply caused by violence and ligament injury as previously thought. The influence of pelvic structures on rotation and vertical stability should be re-evaluated. These findings indicate that we still need to further research ligaments around sacroiliac joint, pelvic floor, and APC pelvic fractures.

Our study also has a number of limitations: First, it was limited to cadaveric biomechanics. Our cadaveric samples are a bit older compared to the pelvis of patients, which may lead to different characteristic. Our load-bearing model does not represent this kind of injury fully. However, when we try to recreate this impact through the restricted and unrestricted models, we find similar results. Second, when the pelvis is under high-energy injury, the loading rate will be slower than expected. Considering the viscoelasticity of biological tissue, particularly the ligaments, the load rate data in our injury model vary. Although these data are controversial, it is generally accepted that with increasing tension, the ultimate load-bearing, stiffness, and energy absorption of ligaments also increase, and ligament failure will be more likely to happen [25, 26]. However, there is no literature regarding the relationship between ligament elongation failure and stress. Although the strain rate of our acute pelvic injury model is not as large as expected, we believe that the anatomical relationship between pelvic ligaments and bone can provide useful information about APC injury [25–27]. The recorded force is not intended to reflect the actual force in pelvic injury. In fact, the distance to the specific ligament failure is the goal of the investigation, which is less affected by viscoelasticity. Third, the quantity of our sample is small; more cadaver specimens will be required to support our hypothesis.

## Conclusion

This work produced the following findings. First, external pelvic rotation injury can be divided into two situations: hemipelvis is restricted and unrestricted, which can result in two different outcomes. When ASL ruptures, the unrestricted group needs greater external rotation angle and force, and SBL or SPL were undamaged, but in the restricted group, both of the two ligaments are injured. In the restricted group, both SBL and SPL were injured, SPL ruptured firstly. Then the SBL and interosseous sacroiliac ligament were damaged while the posterior ligament remained unharmed. In the unrestricted group, the interosseous sacroiliac ligament and posterior sacroiliac ligaments were damaged, while SBL and SPL were not. Second, when ASL fails, pubic symphysis displacement distance has a high fluctuation, ranging from 1.4 to 4.0 cm. The 2.5 cm should not be used as a criterion to distinguish APC type I from APC type II. Third, when ASL is destroyed, we do not observe the inevitable destruction of the pelvic floor ligaments (SPL and SBL).

## Data Availability

The authors confirm that the data supporting the findings of this study are available within the article and its supplementary materials.

## Abbreviations

APC: Anterior posterior compression
ASL: Anterior sacroiliac ligament
SBL: Sacrotuberous ligament
SPL: Sacrospinous ligament
